# Supplementary material for: Are medical outliers associated with worse patient outcomes? A retrospective study within a regional NHS hospital using routine data
Source: BMJ Open. 2017 May 9;7(5):e015676. doi: 10.1136/bmjopen-2016-015676 (PMC5588983; doi:10.1136/bmjopen-2016-015676)
Supplement: Supplementary material [file bmjopen-2016-015676supp001.doc]

STROBE Statement—Checklist of items that should be included in reports of ***cross-sectional studies***

|  | Item No | Recommendation |
| --- | --- | --- |
| **Title and abstract** | 1 | (*a*) Indicate the study’s design with a commonly used term in the title or the abstract  **Within the title (page 1) and study design section of the abstract (page 2 )** |
|  |  | (*b*) Provide in the abstract an informative and balanced summary of what was done and what was found  **See principal findings and conclusion of the abstract (page 2)** |
| Introduction | | |
| Background/rationale | 2 | Explain the scientific background and rationale for the investigation being reported  **See introduction section of paper (page 4-5)** |
| Objectives | 3 | State specific objectives, including any prespecified hypotheses  **See introduction section of paper (page 5)** |
| Methods | | |
| Study design | 4 | Present key elements of study design early in the paper  **Methods section (page 5)** |
| Setting | 5 | Describe the setting, locations, and relevant dates, including periods of recruitment, exposure, follow-up, and data collection  **Methods section (page 5)** |
| Participants | 6 | (*a*) Give the eligibility criteria, and the sources and methods of selection of participants |
| Variables | 7 | Clearly define all outcomes, exposures, predictors, potential confounders, and effect modifiers. Give diagnostic criteria, if applicable  **Methods section (page 6)** |
| Data sources/ measurement | 8* | For each variable of interest, give sources of data and details of methods of assessment (measurement). Describe comparability of assessment methods if there is more than one group |
| Bias | 9 | Describe any efforts to address potential sources of bias  **Methods section (page 6)** |
| Study size | 10 | Explain how the study size was arrived at  **(N/A)** |
| Quantitative variables | 11 | Explain how quantitative variables were handled in the analyses. If applicable, describe which groupings were chosen and why  **Methods section (pages 5-6)** |
| Statistical methods | 12 | (*a*) Describe all statistical methods, including those used to control for confounding  **Methods section (page 6)** |
|  |  | (*b*) Describe any methods used to examine subgroups and interactions |
|  |  | (*c*) Explain how missing data were addressed  **(N/A)** |
|  |  | (*d*) If applicable, describe analytical methods taking account of sampling strategy  **(N/A)** |
|  |  | (*e*) Describe any sensitivity analyses  **(N/A)** |
| Results | | |
| Participants | 13* | (a) Report numbers of individuals at each stage of study—eg numbers potentially eligible, examined for eligibility, confirmed eligible, included in the study, completing follow-up, and analysed |
|  |  | (b) Give reasons for non-participation at each stage |
|  |  | (c) Consider use of a flow diagram  **Results section table 1 (page 7)** |
| Descriptive data | 14* | (a) Give characteristics of study participants (eg demographic, clinical, social) and information on exposures and potential confounders  **Results section table 1 (page 7)** |
|  |  | (b) Indicate number of participants with missing data for each variable of interest  **(N/A)** |
| Outcome data | **15******* | **Report numbers of outcome events or summary measures**  **Results section Table 2 (page 8)** |
| Main results | 16 | (*a*) Give unadjusted estimates and, if applicable, confounder-adjusted estimates and their precision (eg, 95% confidence interval). Make clear which confounders were adjusted for and why they were included  **Results section/ tables 2 & 3** |
|  |  | (*b*) Report category boundaries when continuous variables were categorized  **(N/A)** |
|  |  | (*c*) If relevant, consider translating estimates of relative risk into absolute risk for a meaningful time period  **(N/A)** |
| Other analyses | 17 | Report other analyses done—eg analyses of subgroups and interactions, and sensitivity analyses  **(N/A)** |
| Discussion | | |
| Key results | 18 | Summarise key results with reference to study objectives  **Discussion section first paragraph (page )** |
| Limitations | 19 | Discuss limitations of the study, taking into account sources of potential bias or imprecision. Discuss both direction and magnitude of any potential bias  **Discussion section of the paper/Limitation sub-section (page 10)** |
| Interpretation | 20 | Give a cautious overall interpretation of results considering objectives, limitations, multiplicity of analyses, results from similar studies, and other relevant evidence  **Discussion and conclusion section of paper (pages 9-11)** |
| Generalisability | 21 | Discuss the generalisability (external validity) of the study results  **Discussion section of the paper (page 11)** |
| Other information | | |
| Funding | 22 | Give the source of funding and the role of the funders for the present study and, if applicable, for the original study on which the present article is based  **Within acknowledgements (page 12)** |

*Give information separately for exposed and unexposed groups.

**Note:** An Explanation and Elaboration article discusses each checklist item and gives methodological background and published examples of transparent reporting. The STROBE checklist is best used in conjunction with this article (freely available on the Web sites of PLoS Medicine at http://www.plosmedicine.org/, Annals of Internal Medicine at http://www.annals.org/, and Epidemiology at http://www.epidem.com/). Information on the STROBE Initiative is available at www.strobe-statement.org.
