# Supplementary material for: Are medical outliers associated with worse patient outcomes? A retrospective study within a regional NHS hospital using routine data
Source: BMJ Open. 2017 May 9;7(5):e015676. doi: 10.1136/bmjopen-2016-015676 (PMC5588983; doi:10.1136/bmjopen-2016-015676)
Supplement: Author's manuscript [file bmjopen-2016-015676.draft_revisions.pdf]

BMJ Open

Are medical outliers associated with worse patient outcomes? A retrospective study within a regional NHS hospital using routine data

Journal:	*BMJ Open*
Manuscript ID	bmjopen-2016-015676
Article Type:	Research
Date Submitted by the Author:	22-Dec-2016
Complete List of Authors:	Stylianou, Neophytos; University of Bath, School of Management; Royal United Hospital Bath NHS Trust<br>Fackrell, Robin; Royal United Hospital Bath NHS Trust<br>Vasilakis, C; University of Bath, School of Management
**Primary Subject Heading**:	Health services research
Secondary Subject Heading:	Epidemiology, Health informatics, Medical management
Keywords:	Length of stay, Medical Outlier, Mortality, Quality in health care < HEALTH SERVICES ADMINISTRATION & MANAGEMENT, Patient Outcomes

SCHOLARONE™
Manuscripts

For peer review only

Are medical outliers associated with worse patient outcomes? A retrospective study within a regional NHS hospital using routine data

First Author: Neophytos Stylianou, Centre for Health Innovation & Improvement (Chi^2), School of Management, University of Bath, UK & Researcher-in-Residence, Royal United Hospitals NHS Foundation Trust, Bath, UK.

Author 2: Robin Fackrell, Medical Division, Royal United Hospitals NHS Foundation Trust, Bath, UK.

Author 3: Christos Vasilakis, Centre for Health Innovation & Improvement (Chi^2), School of Management, University of Bath, Bath, UK.

Author order: Neophytos Stylianou; Robin Fackrell; Christos Vasilakis

Contact for correspondence:

Neophytos Stylianou

neophytos.stylianou@hotmail.co.uk

n.stylianou@bath.ac.uk

Word count: 3,227

For peer review only

Abstract

Objective

To explore the quality and safety of patients' healthcare provision by identifying whether being a medical outlier is associated with worse patient outcomes. A medical outlier is a hospital in-patient who is classified as a medical patient for an episode within a spell of care, and has at least one non-medical ward placement within that spell.

Data sources

Secondary data from the Patient Administration System of a district general hospital were provided for the financial years 2013/14-2015/16. The data included 71,038 medical patient spells for the three year period.

Study design

This research was based on a retrospective cross-sectional observational study design. Multivariate logistic regression and zero truncated negative binomial regression were used to explore patient outcomes (in-hospital mortality, 30-day mortality, readmissions and length of stay) while adjusting for several risk factors.

Principal findings

Univariate analysis indicated that an outlying medical in-hospital patient has higher odds for readmission, double the odds of staying longer in the hospital but not significant difference in the odds of in-hospital and 30-day mortality. Multivariate analysis indicates that being a medical outlier does not affect mortality outcomes or re-admission but it does prolong length of stay in hospital.

Conclusions

After adjusting for other factors, medical outliers are associated with an increased LOS while mortality or re-admissions are not worse than patients treated in appropriate specialty wards. This is in line with existing, but limited literature that such patients experience worse patient outcomes. Hospitals need to revisit their policies regarding outlying patients since increased length of stay is associated with increased likelihood of harm events, worse quality of care and increased healthcare costs.

Keywords

Outlier, Mortality, LOS, Quality

For peer review only

Strengths & Limitations

- Evidence associating medical outliers, with patient outcomes are scarce
- This is the first quantitative study in England's NHS that has sought to investigate potential associations between medical outliers and patient outcomes.
- Partial adjustment for patient-specific factors indicates that medical outliers are only associated with longer length of stay.
- Single centre study but having big numbers increases the power of the study
- Routinely collected data limit the association adjustment process but on the other hand makes this study feasible and efficient

For peer review only

Introduction

Faced with ever increasing pressures and targets, hospital bed managers often resort to placing patients on wards that are not specifically designed or designated for the type of care the patient requires. Although patients may be admitted to a ward faster and beds across the entire hospital are used more, patients may not receive treatment in the way it was designed for or by the nurses that are specialised in their care. This phenomenon is commonly known as 'outliers' but it can also be referred to in the literature as 'outlying hospital in-patients', 'overflow', 'sleep-outs' or 'boarders'.[1,2]

Hospital wards are broadly categorised as medical or surgical with each category having a number of generic and specialised wards hosting the delivery of care for patients with similar diagnoses.[3] Examples include stroke patients being cared for in an acute stoke ward and patients who underwent open heart surgery in a cardiac surgery ward. The goal is that by clustering clinical and nursing skills and specialised equipment in a single point in the hospital, i.e. the specialist ward, the clinical management of patients improves and provides an environment that is purposefully designed to meet the clinical and non-clinical needs of patients.[4] By placing patients on a ward designed to treat their condition, the expectation is that their clinical needs are better met.[3]

Nursing and clinical staff providing care for outlying patients are faced with several challenges. The care provided may not be the most appropriate or as timely as it could be, as patients are placed on wards with staff that do not have specific expertise for the patients' condition. Some empirical evidence on medical outliers have suggested that patients who are chosen to be medical outliers are more medically "fit".[5–8] Implicitly, patients that are classified as "fit" to be outliers may be perceived by staff to be of lower priority.[9] The presence of medical outliers may mean longer ward rounds for the clinical teams and thus contributing to valuable clinical time being wasted.[10] Another empirical study in an Australian hospital showed that medical outliers have a higher frequency of emergency calls leading to higher workload on staff who do not know the patients as well, leading to suboptimal decisions which might, in turn, negatively impact upon safety.[9]

This placement of patients in inappropriate care wards leads to a fractured provision of care while compromising continuity of care, another fundamental aspect of high quality care. According to a report from the King's Fund in 2012 which looked at the continuity of care for elderly patients, it showed that patients are frequently moved around from bed to bed within a ward and to different wards.[11] These movements often happened out-of-hours without the patient being informed of the reasons for their move having a detrimental effect on patient experience potentially unpleasant and stressful.[11] This, compromises continuity of care, a fundamental aspect to high quality care,

For peer review only

which is based on establishing relationships with ward staff (continuity of relationship) and their medical management (continuity of management).[11,12]

The phenomenon of medical outliers is more common in publicly funded health systems. It has been observed in England, Wales, Spain, France, Italy, Sweden, Australia, and New Zealand but, as it has not been extensively researched there is scarce good quality evidence available.[1,2,9,13–19] In England's NHS it is an issue often documented in internal hospital reports with some organisations having put in place standard operating procedures and process pathways indicating when a patient can be considered an outlier and what process to be followed.[6–8,20,21] In recent years, two qualitative studies on medical outliers in the NHS highlighted the problem and raised the level of concern of NHS staff regarding patient safety.[1,2] The lack of quantitative studies in this area may be due to the fact that the relevant information is not routinely recorded or collected by hospital information systems or national hospital databases.

Thus, our aim was to investigate potential associations between medical outliers and patient outcomes using routinely collected hospital data. Our working hypothesis is that outlier patients are associated with worse patient outcomes. To the best of our knowledge, this is the first quantitative study in UK that investigated the effects medical outliers have on patient outcomes regardless of diagnosis.

Methods

Data for this retrospective observational study were provided by the Business Intelligence Unit of the Royal United Hospitals Bath NHS Foundation Trust, a district general hospital in South West England which serves a population of 550,000 people with approximately 565 beds. The time period of the study covered three financial years, 2013/14-2015/16. A medical outlier was defined as a hospital in-patient who was classified as a medical patient for an episode within a spell of care, and had at least one ward placement on a non-medical ward within that spell. An episode of care was the time a patient spent under the care of one consultant. Many episodes comprised a spell of care which was the continuous, usually uninterrupted, stay of a patient within a hospital provider.[22]

The data were provided in a hierarchical structure as follows: i) Moves within episodes, ii) Episodes of care, and iii) Spell level record. We used this data structure to establish whether a patient had been classified as an outlier at any point within their spell of care at the hospital. After establishing if each patient spent time outlying we used the dominant episode data for further analysis. A dominant episode is usually the first episode in a multi episode spell which contains the primary

For peer review only

procedure and if no primary procedure exists then it is the first episode that contains the primary diagnosis for the spell.[23]

The patient outcomes investigated were in-hospital mortality, 30-day mortality (death within 30 days of discharge), readmission to the same hospital within 30 days of discharge and hospital length of stay.

Multivariate logistic regression was used for readmission and mortality outcomes and zero truncated negative binomial regression was used for length of stay examination. Confounding factors that were adjusted for were age, sex, primary diagnosis ICD category (of the dominant episode), admission source, admission method (emergency or not), discharge method, discharge destination, count of procedures, count of "secondary" diagnosis, weekend admission, weekend discharge and socioeconomic status of the patient. Socioeconomic status was measured using the 2015 Index of Multiple Deprivation (IMD) score at Lower Super Output Areas (LSOA). IMD is a measure of deprivation calculated using seven domains of deprivation.[24] LSOAs are small geographical areas of roughly equal population size, averaging 1,500 residents or 650 households.[25] The smaller the score the more deprived an area is. All analyses were performed using Stata 13.1.[26]

Results

General description of the data

A full extract of the Patient Administration System of the hospital was provided for the financial years 2013/14/-2015/16. This extract was made up of 71,038 dominant spells in which patients were classified as medical cases (as opposed to surgical or maternity). A general description of the data is shown on Table 1. Age, sex, secondary diagnoses, procedures counts did not present significant differences. Mean (standard deviation, SD) age of patients admitted to hospital was 66.40 (20.66) and the age distribution for medical outliers and non-outliers was similar. Male spells accounted for 47.61% of the total admitted spells and the remaining 52.39% were women. In-hospital mortality was roughly 5% of all spells for the three years of the data and 30-day mortality was 3.26%. Of the 71,038 spells of care, 9.88% have been outlying at some point of their care. Approximately 23% of the spells were weekend admissions with 14.10% outlying. IMD ranged from 32 to 32812 and the mean (SD) was 20,342.4 (7,991.13). Medical outliers and non-outliers spells had relatively similar IMD scores.

On average, patient spells had seven secondary diagnoses attached to their medical record and this was similar in both outlier groups. The same applied for procedure numbers but those were on average three. Regarding the overall primary diagnosis the most common was signs and symptoms

For peer review only

which do not give a definitive diagnosis. This was followed closely by diseases of the circulatory system. The primary diagnosis was different between non-medical outliers and medical outliers. For non-medical outliers the most common primary diagnosis was diseases of the circulatory system followed closely by category symptoms and signs and abnormal clinical findings not elsewhere classified, whereas for medical outliers the most common primary diagnosis category was diseases of the respiratory system followed by diseases of the digestive system.

Table 1: General description of the data

Characteristic		Non-outlying	Out-lying	Total
Spells		64,017 (90.12)	7,021 (9.88)	71,038
Gender	Male	30,487 (90.14)	3,333 (9.86)	33,820
	Female	33,530 (90.09)	3,688 (9.91)	37,218
Age*		66.16(20.64)	68.62 (20.62)	66.40 (20.66)
Secondary Diagnosis numbers*		7.17 (3.74)	7.85(3.83)	7.24 (3.76)
Procedure numbers*		3.20 (2.41)	3.93 (2.97)	3.29 (2.49)
Weekend admissions		13,861 (85.90)	2,276 (14.10)	16,137
IMD*		20,360.08 (7,992.15)	20,181.38 (7,980.63)	20,342.4 (7,991.13)
Primary diagnosis ICD-10 category	Certain infectious and parasitic diseases	2,470 (3.86)	352 (5.01)	2,822 (3.97)
	Neoplasms	2,460 (3.84)	381 (5.43)	2,841 (4.00)
	Diseases of the blood and blood-forming organs and certain disorders involving the immune mechanism	760 (1.19)	68 (0.97)	8289 (1.17)
	Endocrine, nutritional and metabolic diseases	1,521 (2.38)	146 (2.08)	1,667 (2.35)
	Mental and Behavioural disorders	1,426 (2.23)	117 (1.67)	1,543 (2.17)
	Diseases of the nervous system	2,236 (3.49)	118 (1.68)	2,354 (3.31)
	Diseases of the eye and adnexa	137 (0.21)	11 (0.16)	148 (0.21)
	Diseases of the ear and mastoid process	206 (0.32)	32 (0.46)	238 (0.34)
	Diseases of the circulatory system	12,283 (19.19)	522 (7.43)	12,805 (18.03)
	Diseases of the respiratory system	9,139 (14.28)	1,277 (18.19)	10,416 (14.66)
	Diseases of the digestive system	2,934 (4.58)	1,022 (14.56)	3,956 (5.57)
	Diseases of the skin and subcutaneous tissue	1,013 (1.58)	276 (3.93)	1,289 (1.81)
	Diseases of the musculoskeletal system and connective tissue	2,058 (3.21)	351 (5.00)	2,409 (3.39)
	Diseases of the genitourinary system	3,019 (4.72)	671 (9.56)	3,690 (5.19)

	Pregnancy, childbirth and the puerperium	109 (0.17)	29 (0.41)	138 (0.19)
	Congenital malformations, deformations and chromosomal abnormalities	19 (0.03)	2 (0.03)	21 (0.03)
	Symptoms, signs and abnormal clinical and laboratory findings, not elsewhere classified	12,026 (18.79)	852 (12.14)	12,878 (18.13)
	Injury, poisoning and certain other consequences of external causes	9,970 (15.57)	777 (11.07)	10,747 (15.13)
	Factors influencing health status and contact with health services	215 (0.34)	16 (0.23)	231 (0.33)
	Unknown	16 (0.02)	1 (0.01)	17 (0.02)

All values are numbers (percentages) unless otherwise stated
*Mean (SD)

Medical Outliers and patients outcomes

Table 2 presents the patient outcomes for both medical and non-medical outliers. Approximately 10% of the in-hospital mortality occurred to medical outliers. A similar percentage was observed for 30-day mortality. Of all readmissions to the hospital, approximately 11% occurred by patients that were medical outliers at some point during their care. Univariate analysis of medical outliers on the patient outcome revealed that medical outliers do not affect in-hospital or 30-day mortality but they do affect the readmission probabilities with statistically significant odds ratio (95% CI) of 1.186 (1.105 – 1.272).

Table 2: Patient outcomes and medical outliers

Characteristic	Non-outlying	Out-lying	Effect size (95% CI)**
In-hospital mortality	3,187 (90.26)	344 (9.74)	0.983 (0.877 -1.102)
30-day mortality	2,072 (89.50)	243 (10.50)	1.071 (0.936 – 1.227)
Readmission	8,107 (88.73)	1,030 (11.27)	1.186 (1.105 – 1.272)
LOS*	3(7)	7(7)	0.672 (0.624 – 0.719)

All values are numbers (percentages) unless otherwise stated
*Median (IQR)
**Odds ratio with the exception of LOS that the negative binomial regression coefficient is given

Table 2 shows that outliers have more than double the LOS of non-medical outlier spells. As the distribution of LOS is highly positively skewed, median (IQR) is reported. LOS is further described in Figure 1 where the distribution of both spell groups is shown. It is clear that medical outliers are staying in the hospital for longer periods of time and more patient spells have recorded more than

For peer review only

30 days in hospital compared to non-medical outliers. Univariate analysis for LOS indicated that the log count for medical outlier patients is 0.672 more than non-medical outliers, equating to staying in hospital approximately two days longer than non-medical outliers.

<<Figure 1>>

After adjusting for variables that may affect the patient outcomes we were investigating, it was found that medical outliers are not associated with increased odds of in-hospital mortality, 30 day mortality or readmission, Table 3. However, medical outliers were found to be associated with an increasing effect on LOS. Medical outliers compared to non-medical outliers, while adjusting for all other variables and keeping them constant the model, have a log count of stay of 0.042 higher compared to non-medical outliers. Keeping all other variables constant and the continuous variables of the multivariable models at their mean values indicated that the expected LOS for non-medical outliers was 3.86 (95% CI 3.22 to 4.50) while for medical outliers was 4.03 (95% CI 3.36 to 4.7). In other words, medical outliers spent 0.17 days (p-value 0.006) more in hospital compared to non-medical outliers when other variables are kept constant or at their mean levels.

Table 3: Effect sizes of medical outliers on patient outcomes adjusting for other independent variables in the multivariable models

Patient Outcome	Effect size*	Significance (0.05)	(95% CI)
In-hospital Mortality	0.679	<0.001	0.600 - 0.769
30-day mortality	0.711	<0.001	0.614 - 0.822
Readmission	0.918	0.052	0.843 - 1.001
LOS	0.042	0.004	0.014 - 0.071

*Odds ratio with the exception of LOS that the zero truncated negative binomial coefficient is given

Discussion

Our analysis showed that approximately 10% of the patients classified as medical patients were outliers at least once during their entire hospitalisation. The results indicated that age, comorbidity, emergency admissions within the same year, primary diagnosis category and weekend admission impose significant effects on in-hospital mortality, 30-day mortality, readmissions and LOS. These factors are known to affect the patient outcomes we used in this study. Being a medical outlier does not appear to affect mortality, either in-hospital or 30-day after discharge. This was also observed after controlling for confounding variables. The preventive odds observed for mortality outcomes during the multivariable analysis can be explained by the fact that patients classified based on

For peer review only

medical expertise as being more severely affected by their disease/condition, are kept on their home wards and patients that become outliers are typically less unwell.

Being a medical outlier is associated with higher odds of being readmitted to the same hospital within 30 days but after partially adjusting for a number of confounding variables this association was diluted. However, we found that outlier patients have double the length of stay of non-outlying patients and multivariable analysis confirmed this increase in length of stay.

Our results are in-line with those found in the extant literature comprising a limited number of studies. Specifically, Alameda and Suarez in a 2009 study performed in Spain found that readmissions were not affected by being a medical outlier but that there was an increasing effect on LOS.[15] Longer LOS for outlying patients was also observed by Stowell et al. in a French study in 2013.[18]

The increased length of stay medical outliers have compared to non-medical outliers could be explained by the difference in primary disease diagnosis. As stated previously the primary diagnosis differed between the two groups with outliers being predominantly diagnosed with circulatory and respiratory diseases whereas non-outliers were primarily diagnosed with circulatory diseases and symptoms that could not be classified. Length of stay is an important metric of quality of healthcare provision as it is often associated with complications occurring within the hospital.[27–29] Further reasons why medical outlying patients experience longer length of stays include the delayed medical contact between arrival and first examination in the case the patient was placed straight away on an "inappropriate" ward, insufficient contact with medical and nursing personnel during outlying period and also inability of nursing personnel to meet patient needs because of lack of specialisation on the disease/condition of the patient.

The field of medical outliers is not extensively researched although evidence does exist to suggest that being in the right bed at the right time is beneficial to patients as a number of studies in stroke, burns, asthma and gastrointestinal haemorrhage has shown. [30–33] However, there is not enough evidence in the literature to give a definitive answer on the effect of medical outliers on patient outcomes. This is because some studies were only looking at qualitative data to understand what causes medical outliers, or because the quantitative studies were based on small sample sizes with a lot of exclusion criteria or performed in a single hospital department or ward. Our study is the first to analyse the effect medical outliers have on patient outcomes using a large set of routinely collected data.

For peer review only

Limitations

Limitations are inevitable in retrospective, observational studies. Our study used routinely collected data from the hospital's patient administration system, which is known to be a limiting factor in studies. The secondary data usage is of limited use for confounding factors as we could not adjust for factors we believed would be important in the multivariable models we developed. For example we could not adjust for severity of patient although we used the proxies of number of procedures and diagnosis codes recorded in the patient record for that spell. A prospective study, could allow for the collection of specific clinical data such as severity of the patient and other factors associated with patient movement and outlier status such as time to examine patients on ward rounds or patient complications but this would need a lot of resources to be able to get as many data.

Out study is a single centre study which in theory minimises generalisability although the amount of routinely collected data we used somewhat mitigates this limitation. Given the lack of relevant data being recorded in national datasets such as Hospital Episode Statistics (HES) only a study which includes prospective data collection method could overcome this limitation fully.

Reasons for longer patient LOS could not be investigated as they are not typically recorded in administrative databases. We could not identify nursing and medical staff numbers per ward, experience of nurses and doctors, delay in prescription of drugs or laboratory analyses and mistakes occurring during prescription or diagnosis. These could be further investigated to elucidate what makes outlying patients more susceptible to different patient outcomes compared to non-outlying patients.

Longer length of stay in hospital makes the patients susceptible to hospital acquired infections and could be further investigated. Again, infection data are not routinely collected for every patient admission.

Finally, our study investigated medical outliers only and not surgical or maternity cases. A recent publication by The Royal College of Surgeons of England showed that surgical patients too experience outlying, which raises the same concerns regarding patient safety, continuity of care and overall levels of provision of high quality healthcare.[10]

Conclusions

Patient outcomes are directly associated with quality of care. We studied one factor which is associated with quality of care and found that the physical location of a medical in-patient in the hospital is associated with longer length of stay. In busy general hospitals the phenomenon of patients placed on different wards through the hospital is not a rare event. Unlike previous studies

For peer review only

on this issues, our study was not limited to a single clinical service. Despite its limitations, our study findings can be used to inform. We showed that placing patients in different specialty wards does have a direct effect on patient outcomes. However, we do not advise on eliminating the practice since that may mean more time spent waiting for a bed, for example, in an already busy A&E department or patients having their elective admission cancelled due to bed unavailability. A better usage of our findings would be informing hospital managers and clinicians of the negative effects associated with medical outliers and these are taken into account when devising hospital policies and procedures, or when deciding on an ad-hoc basis. As it is common with many secondary data analysis studies, more research is needed to investigate the effects more thoroughly (for example, through a multi-centre study of prospectively collected data). The call for additional research is particularly strong in the phenomenon investigated here as the practice of placing patients on any available hospital bed is wide spread and the implications on patient outcomes and quality of care not well known or understood.

Acknowledgments/ Funding

The Researchers-in-Residence programme of research is funded by the Royal United Hospitals NHS Foundation Trust.

Conflict of interest

We would like to declare that Robin Fackrell is the Head of Medical Division at the RUH Bath. Christos Vasilakis and Neophytos Stylianou both received funding form Royal United Hospitals of Bath NHS Trust to perform this research as part of the Researchers-in-Residence programme.

Contributory statement

NS, RF and CV conceived the idea for the study. NS and CV designed the study. NS analysed all data and wrote the original manuscript with input from CV. RF facilitated the acquisition of data and provided clinical expert guidance. All authors commented on the manuscript.

Data sharing statement

Data were provided by the Business Intelligence Unit of Royal United Hospitals of Bath. As we cannot keep the data based on data sharing agreements in place, data can be requested by the provider if data sharing agreement requirements are met by recipient.

References

1 Goulding L, Adamson J, Watt I, *et al.* Lost in hospital: A qualitative interview study that

For peer review only

explores the perceptions of NHS inpatients who spent time on clinically inappropriate hospital wards. *Heal Expect* 2015;**18**:982–94. doi:10.1111/hex.12071

2 Goulding L, Adamson J, Watt I, *et al.* Patient safety in patients who occupy beds on clinically inappropriate wards: a qualitative interview study with NHS staff. *BMJ Qual Saf* 2012;**21**:218–24. doi:10.1136/bmjqs-2011-000280

3 Audit Commission. Bed management. London: 2003.

4 Wright N, Shapiro LM, Nicholson J. Treatment of renal failure in a non-specialist unit. *Br Med J* 1980;**281**:117–8.http://www.ncbi.nlm.nih.gov/pubmed/7427208 (accessed 4 Aug2016).

5 Bulman R. Bed Management Policy. Salfrod Royla NHS Foundation Trust. Salford: 2016.

6 Cullen S, OArk C, Jones L, *et al.* Standard Operating Procedure for Outlying Patients. University Hospitals of Morecambe Bay NHS Foundation Trust. Kendal: 2015.

7 Martin N. Adult Outliers Policy. Portsmouth Hospitals NHS Trust. Portsmouth: 2015.

8 Purse M. Adult Outliers & Buffer Capacity Policy. Portsmouth Hospitals NHS Trust. Portsmouth: 2009.

9 Santamaria JD, Tobin AE, Anstey MH, *et al.* Do outlier inpatients experience more emergency calls in hospital? An observational cohort study. *Med J Aust* 2014;**200**:45–8. doi:10.5694/mja12.11680

10 Williamson M, Ghazaly M, Bhatt N. Looking after patients, not looking for patients. *Bull R Coll Surg Engl* 2015;**97**:260–2. doi:10.1308/147363515X14272809070960

11 Cornwell J, Levenson R, Sonola L, *et al.* Continuity of care for older hospital patients A call for action. The King's Fund. London: 2012.

12 Freeman G, Hughes J. Continuity of care and the patient experience. The King's Fund. London: 2010.

13 Lloyd JM, Elsayed S, Majeed A, *et al.* The practice of out-lying patients is dangerous: A multicentre comparison study of nursing care provided for trauma patients. *Injury* 2005;**36**:710–3. doi:10.1016/j.injury.2004.11.006

14 Lepage B, Robert R, Lebeau M, *et al.* Use of a risk analysis method to improve care management for outlying inpatients in a university hospital. *Qual Saf Heal Care* 2009;**18**:441–5. doi:10.1136/qshc.2007.025742

15 Alameda C, Suarez C. Clinical outcomes in medical outliers admitted to hospital with heart failure. *Eur J Intern Med* 2009;**20**:764–7. doi:10.1016/j.ejim.2009.09.010

16 National Audit Office. Inpatient Admissions and Bed Management in NHS acute hospitals. London: 2000.

17 Serafini F, Fantin G, Brugiolo R, *et al.* Outlier admissions of medical patients: prognostic implications of outlying patients. The experience of the Hospital of Mestre. *Ital J Med* 2015;**9**:299–302. doi:10.4081/itjm.2015.528

18 Stowell A, Claret P-G, Sebbane M, *et al.* Hospital out-lying through lack of beds and its impact on care and patient outcome. *Scand J Trauma Resusc Emerg Med* 2013;**21**:17. doi:10.1186/1757-7241-21-17

19 Creamer GL, Dahl A, Perumal D, *et al.* Anatomy of the ward round: the time spent in different activities. *ANZ J Surg* 2010;**80**:930–2. doi:10.1111/j.1445-2197.2010.05522.x

For peer review only

20 Kadum D. Right Patient, Right Place. The Shrewsburt and Telford Hospital NHS Trust. Shrewsbury: 2013.

21 Bryant M, Daly M. Right Patient, Right care, Right Place. Royal Devon and Exeter NHs Foundation Trust. Exeter: 2011.

22 HSCIC. NHS DATA MODEL AND DICTIONARY. http://www.datadictionary.nhs.uk/web_site_content/navigation/main_menu.asp

23 HSCIC. PbR data user guide. Leeds: 2013.

24 English indices of deprivation 2015. 2015.https://www.gov.uk/government/statistics/english-indices-of-deprivation-2015

25 ONS. Super Output Area (SOA). 2016.http://webarchive.nationalarchives.gov.uk/20160105160709/http://www.ons.gov.uk/ons/guide-method/geography/beginner-s-guide/census/super-output-areas--soas-/index.html

26 Stata: Data Analysis and Statistical Software. http://www.stata.com/

27 McAleese P, Odling-Smee W. The effect of complications on length of stay. *Ann Surg* 1994;**220**:740–4.http://www.ncbi.nlm.nih.gov/pubmed/7986140 (accessed 14 Dec2016).

28 Hassan M, Tuckman HP, Patrick RH, *et al.* Hospital length of stay and probability of acquiring infection. *Int J Pharm Healthc Mark* 2010;**4**:324–38. doi:10.1108/17506121011095182

29 Dulworth S, Pyenson B. Healthcare-associated infections and length of hospital stay in the Medicare population. *Am J Med Qual* 2004;**19**:121–7.

30 Sanderson JD, Taylor RF, Pugh S, *et al.* Specialized gastrointestinal units for the management of upper gastrointestinal haemorrhage. *Postgrad Med J* 1990;**66**:654–6.http://www.ncbi.nlm.nih.gov/pubmed/2217034 (accessed 4 Aug2016).

31 Bucknall CE, Robertson C, Moran F, *et al.* Management of asthma in hospital: a prospective audit. *Br Med J (Clin Res Ed)* 1988;**296**:1637–9.http://www.ncbi.nlm.nih.gov/pubmed/3135048 (accessed 4 Aug2016).

32 Mayor S. Stroke patients prefer care in specialist units. *BMJ* 2005;**331**:130. doi:10.1136/bmj.331.7509.130-a

33 Dunn KW. Standards and Strategy for Burn Care. 2001. http://www.ibidb.org/downloads-mainmenu-28/cat_view/14-general-reports (accessed 14 Oct2011).

Figures

Figure 1: Distribution of LOS for medical outlier and non-medical outliers

For peer review only

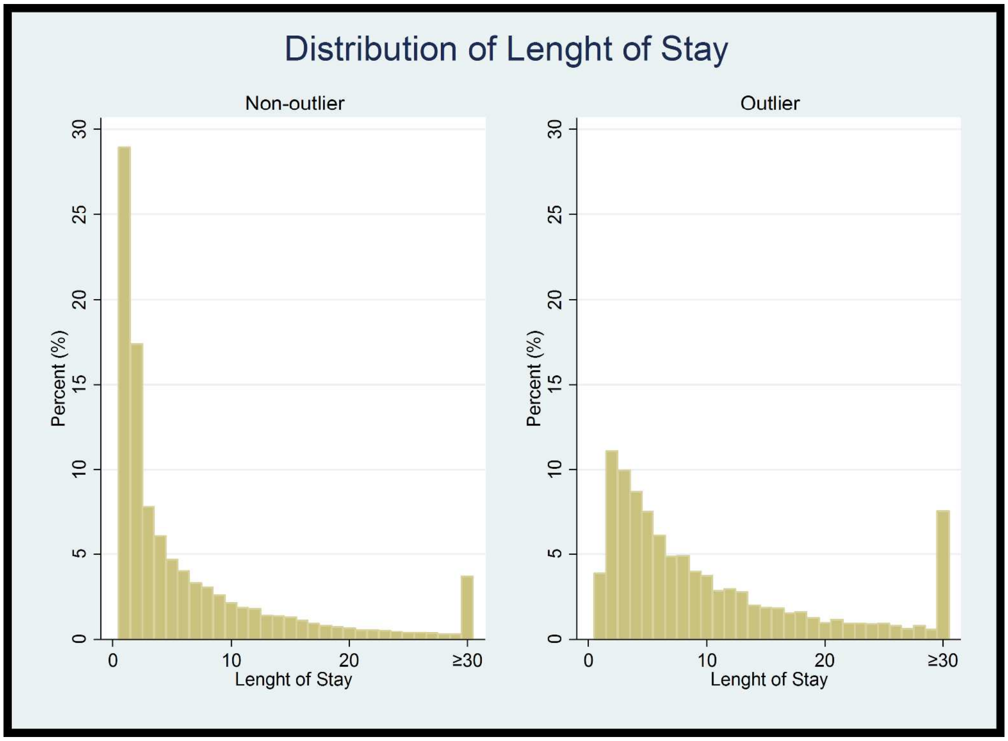

Figure 1: Distribution of LOS for medical outlier and non-medical outliers

887x650mm (96 x 96 DPI)

For peer review only

BMJ Open

Are medical outliers associated with worse patient outcomes? A retrospective study within a regional NHS hospital using routine data

Journal:	*BMJ Open*
Manuscript ID	bmjopen-2016-015676.R1
Article Type:	Research
Date Submitted by the Author:	07-Mar-2017
Complete List of Authors:	Stylianou, Neophytos; University of Bath, School of Management; Royal United Hospital Bath NHS Trust<br>Fackrell, Robin; Royal United Hospital Bath NHS Trust<br>Vasilakis, C; University of Bath, School of Management
**Primary Subject Heading**:	Health services research
Secondary Subject Heading:	Epidemiology, Health informatics, Medical management
Keywords:	Length of stay, Medical Outlier, Mortality, Quality in health care < HEALTH SERVICES ADMINISTRATION & MANAGEMENT, Patient Outcomes

SCHOLARONE™
Manuscripts

For peer review only

Are medical outliers associated with worse patient outcomes? A retrospective study within a regional NHS hospital using routine data

First Author: Neophytos Stylianou, Centre for Health Innovation & Improvement (Chi^2), School of Management, University of Bath, UK & Researcher-in-Residence, Royal United Hospitals NHS Foundation Trust, Bath, UK.

Author 2: Robin Fackrell, Medical Division, Royal United Hospitals NHS Foundation Trust, Bath, UK.

Author 3: Christos Vasilakis, Centre for Health Innovation & Improvement (Chi^2), School of Management, University of Bath, Bath, UK.

Author order: Neophytos Stylianou; Robin Fackrell; Christos Vasilakis

Contact for correspondence:

Neophytos Stylianou

n.stylianou@bath.ac.uk

Word count: 3,450

For peer review only

Abstract

Objective

To explore the quality and safety of patients' healthcare provision by identifying whether being a medical outlier is associated with worse patient outcomes. A medical outlier is a hospital in-patient who is classified as a medical patient for an episode within a spell of care, and has at least one non-medical ward placement within that spell.

Data sources

Secondary data from the Patient Administration System of a district general hospital were provided for the financial years 2013/14-2015/16. The data included 71,038 medical patient spells for the three year period.

Study design

This research was based on a retrospective cross-sectional observational study design. Multivariate logistic regression and zero truncated negative binomial regression were used to explore patient outcomes (in-hospital mortality, 30-day mortality, readmissions and length of stay) while adjusting for several confounding factors.

Principal findings

Univariate analysis indicated that an outlying medical in-hospital patient has higher odds for readmission, double the odds of staying longer in the hospital but no significant difference in the odds of in-hospital and 30-day mortality. Multivariable analysis indicates that being a medical outlier does not affect mortality outcomes or re-admission but it does prolong length of stay (LOS) in hospital.

Conclusions

After adjusting for other factors, medical outliers are associated with an increased LOS while mortality or re-admissions are not worse than patients treated in appropriate specialty wards. This is in line with existing, but limited literature that such patients experience worse patient outcomes. Hospitals may need to revisit their policies regarding outlying patients since increased length of stay is associated with an increased likelihood of harm events, worse quality of care and increased healthcare costs.

Keywords

Outlier, Mortality, LOS, Quality

For peer review only

Strengths & Limitations

- There is scarce evidence associating medial outliers and patient outcomes.
- This is the first quantitative study in England's NHS that has sought to investigate potential associations between medical outliers and patient outcomes.
- Adjusting for several patient-specific confounding factors, indicates that medical outliers are only associated with longer length of stay.
- Large dataset used increases the power of the study and minimises the single centre limitation.
- Routinely collected data limit the association adjustment process, but on the other hand makes this study feasible and efficient

For peer review only

Introduction

Faced with ever increasing pressures and targets, hospital bed managers often resort to placing patients on wards that are not specifically designed or designated for the type of care the patient requires. Although patients may be admitted to a ward faster and beds across the entire hospital are used more, patients may not receive treatment in the way it was designed for or by the nurses that are specialised in their care. This phenomenon is commonly known as 'outliers' but it can also be referred to in the literature as 'outlying hospital in-patients', 'overflow', 'sleep-outs' or 'boarders'.[1,2]

Hospital wards are broadly categorised as medical or surgical with each category having a number of generic and specialised wards hosting the delivery of care for patients with similar diagnoses.[3] Examples include stroke patients being cared for in an acute stoke ward and patients who underwent open heart surgery in a cardiac surgery ward. The goal is that by clustering clinical and nursing skills and specialised equipment in a single point in the hospital, i.e. the specialist ward, the clinical management of patients improves and provides an environment that is purposefully designed to meet the clinical and non-clinical needs of patients.[4] By placing patients on a ward designed to treat their condition, the expectation is that their clinical needs are better met.[3]

Nursing and clinical staff providing care for outlying patients are faced with several challenges. The care provided may not be the most appropriate or as timely as it could be, as patients are placed on wards with staff that do not have specific expertise for the patients' condition.[1,2] Some empirical evidence on medical outliers have suggested that patients who are chosen to be medical outliers are more medically "fit".[5–8] Implicitly, patients that are classified as "fit" to be outliers may be perceived by staff to be of lower priority.[9] The presence of medical outliers may mean longer ward rounds for the clinical teams and thus contributing to valuable clinical time being wasted.[10] Another empirical study in an Australian hospital showed that medical outliers have a higher frequency of emergency calls leading to higher workload on staff who do not know the patients as well, leading to suboptimal decisions which might, in turn, negatively impact upon safety.[9]

This placement of patients in care wards which do not offer the specialised care the patient needs, may lead to a suboptimal and fractured provision of care. Continuity of care, a fundamental aspect of high quality care, is based on establishing relationships with ward staff (continuity of relationship) and their medical management (continuity of management).[11,12]. A recent report which looked at continuity of care for elderly patients, showed that patients are frequently moved around from bed to bed within a ward and to different wards.[11] These movements, often happening out-of-hours

For peer review only

and without the patient being informed of the reasons, have shown to be potentially unpleasant and stressful , have a detrimental effect on patient experience and compromise continuity of care.[11]

The phenomenon of medical outliers is more common in publicly funded health systems.[13] It has been observed in England, Wales, Spain, France, Italy, Sweden, Australia, and New Zealand but, as it has not been extensively researched there is scarce good quality evidence available.[1,2,9,13–21] In England's NHS it is an issue often documented in internal hospital reports with some organisations having put in place standard operating procedures and process pathways indicating when a patient can be considered an outlier and what process to be followed.[6–8,22,23] In recent years, two qualitative studies on medical outliers in the NHS highlighted the problem and raised the level of concern of NHS staff regarding patient safety.[1,2] The lack of quantitative studies in this area may be due to the fact that the relevant information is not routinely recorded or collected by hospital information systems or national hospital databases.

Thus, our aim was to investigate potential associations between medical outliers and patient outcomes using routinely collected hospital data. Our working hypothesis is that outlier patients are associated with worse patient outcomes. To the best of our knowledge, this is the first quantitative study in UK that investigated the effects medical outliers have on patient outcomes regardless of diagnosis.

Methods

Data for this retrospective observational study were provided by the Business Intelligence Unit of the Royal United Hospitals Bath NHS Foundation Trust, a district general hospital in South West England which serves a population of 550,000 people with approximately 565 beds. The time period of the study covered three financial years, 2013/14-2015/16. Since this study used anonymised, non-identifiable secondary data an ethics approval was not necessary according to the NHS regulations.[24] A medical outlier was defined as a hospital in-patient who was classified as a medical patient for an episode within a spell of care, and had at least one ward placement on a non-medical ward within that spell. An episode of care was the time a patient spent under the care of one consultant. Many episodes comprised a spell of care which was the continuous, usually uninterrupted, stay of a patient within a hospital provider.[25]

The data were provided in a hierarchical structure as follows: i) patient ward moves within episodes of care, ii) patient episodes of care, and iii) patient spells. We used this data structure to establish whether a patient had been classified as an outlier at any point within their spell of care at the hospital. We then used the dominant episode data for further analysis. A dominant episode is the

For peer review only

first episode in a multi episode spell which contains the primary procedure and if no primary procedure exists, then it is the first episode that contains the primary diagnosis for the spell.[26] A schematic illustration of the dataset structure is shown in Figure 1.

<<Figure 1>>

The patient outcomes investigated were in-hospital mortality, 30-day mortality (death within 30 days of discharge), readmission to the same hospital within 30 days of discharge and hospital length of stay. For the patient outcome of readmission, patients that died in hospital were excluded.

Univariate analysis was used to establish any relationship between the patient outcome under investigation when the patient was either an outlier or not. Logistic regression was used for readmission and mortality outcomes and zero truncated negative binomial regression was used for length of stay examination. The multivariable methods were used to adjust for potential confounding factors that are already known to be associated with the outcomes under investigation. Confounding factors that were adjusted for were age, sex, primary diagnosis ICD category (of the dominant episode), admission method (emergency or not), count of procedures, count of "secondary" diagnosis, weekend admission, weekend discharge and socioeconomic status of the patient. Socioeconomic status was measured using the 2015 Index of Multiple Deprivation (IMD) score at Lower Super Output Areas (LSOA). IMD is a measure of deprivation calculated using seven domains of deprivation.[27] LSOAs are small geographical areas of roughly equal population size, averaging 1,500 residents or 650 households.[28] The smaller the score the more deprived an area is. All statistical significance was measured at a p-value of less than 0.05. All analyses were performed using Stata 13.1.[29]

Results

General description of the data

A full extract of the Patient Administration System of the hospital was provided for the financial years 2013/14/-2015/16. This extract was made up of 71,038 dominant spells in which patients were classified as medical cases (as opposed to surgical or maternity). A general description of the data is shown on Table 1. Age, sex, secondary diagnoses, procedures counts did not present significant differences. Mean (standard deviation, SD) age of patients admitted to hospital was 66.40 (20.66) and the age distribution for medical outliers and non-outliers was similar. Male spells accounted for 47.61% of the total admitted spells and the remaining 52.39% were women. In-hospital mortality was roughly 5% of all spells for the three years of the data and 30-day mortality was 3.26%. Of the 71,038 spells of care, 9.88% have been outlying at some point of their care. Approximately 23% of

For peer review only

the spells were weekend admissions with 14.10% outlying. IMD ranged from 32 to 32812 and the mean (SD) was 20,342.4 (7,991.13). Medical outliers and non-outliers spells had relatively similar IMD scores.

On average, patient spells had six secondary diagnoses attached to their medical record. Non outliers had 6.17 and medical outliers had 6.85 a difference which was statistically significant (p<0.001).. The same applied for procedure numbers but those were on average three. Regarding the overall primary diagnosis the most common was signs and symptoms which do not give a definitive diagnosis. This was followed closely by diseases of the circulatory system. The primary diagnosis was different between non-medical outliers and medical outliers. For non-medical outliers the most common primary diagnosis was diseases of the circulatory system followed closely by category symptoms and signs and abnormal clinical findings not elsewhere classified, whereas for medical outliers the most common primary diagnosis category was diseases of the respiratory system followed by diseases of the digestive system.

Table 1: General description of the data

Characteristic		Non-outlying	Out-lying	p-value	Total
Spells		64,017 (90.12)	7,021 (9.88)	-	71,038
Gender	Male	30,487 (90.14)	3,333 (9.86)	0.809	33,820
	Female	33,530 (90.09)	3,688 (9.91)		37,218
Age*		66.16(20.64)	68.62 (20.62)	<0.001	66.40 (20.66)
Secondary Diagnosis numbers*		6.17 (3.74)	6.85(3.83)	<0.001	6.24 (3.76)
Procedure numbers*		1.24 (2.16)	1.81 (2.81)	<0.001	3.29 (2.49)
Weekend admissions		13,861 (85.90)	2,276 (14.10)		16,137
IMD*		20,360.08 (7,992.15)	20,181.38 (7,980.63)	0.079	20,342.4 (7,991.13)
Primary diagnosis ICD-10 category	Certain infectious and parasitic diseases	2,470 (3.86)	352 (5.01)	-	2,822 (3.97)
	Neoplasms	2,460 (3.84)	381 (5.43)		2,841 (4.00)
	Diseases of the blood and blood-forming organs and certain disorders involving the immune mechanism	760 (1.19)	68 (0.97)		8289 (1.17)
	Endocrine, nutritional and metabolic diseases	1,521 (2.38)	146 (2.08)		1,667 (2.35)
	Mental and Behavioural disorders	1,426 (2.23)	117 (1.67)		1,543 (2.17)
	Diseases of the nervous system	2,236 (3.49)	118 (1.68)		2,354 (3.31)
	Diseases of the eye and adnexa	137 (0.21)	11 (0.16)		148 (0.21)
	Diseases of the ear and mastoid process	206 (0.32)	32 (0.46)		238 (0.34)

	Diseases of the circulatory system	12,283 (19.19)	522 (7.43)		12,805 (18.03)
	Diseases of the respiratory system	9,139 (14.28)	1,277 (18.19)		10,416 (14.66)
	Diseases of the digestive system	2,934 (4.58)	1,022 (14.56)		3,956 (5.57)
	Diseases of the skin and subcutaneous tissue	1,013 (1.58)	276 (3.93)		1,289 (1.81)
	Diseases of the musculoskeletal system and connective tissue	2,058 (3.21)	351 (5.00)		2,409 (3.39)
	Diseases of the genitourinary system	3,019 (4.72)	671 (9.56)		3,690 (5.19)
	Pregnancy, childbirth and the puerperium	109 (0.17)	29 (0.41)		138 (0.19)
	Congenital malformations, deformations and chromosomal abnormalities	19 (0.03)	2 (0.03)		21 (0.03)
	Symptoms, signs and abnormal clinical and laboratory findings, not elsewhere classified	12,026 (18.79)	852 (12.14)		12,878 (18.13)
	Injury, poisoning and certain other consequences of external causes	9,970 (15.57)	777 (11.07)		10,747 (15.13)
	Factors influencing health status and contact with health services	215 (0.34)	16 (0.23)		231 (0.33)
	Unknown	16 (0.02)	1 (0.01)		17 (0.02)

All values are numbers (percentages) unless otherwise stated
T-tests and Chi-square tests were used to compare continuous and categorical variables respectively
*Mean (SD)

Medical Outliers and patients outcomes

Table 2 presents the patient outcomes for both medical and non-medical outliers. There were 3,531 in-hospital deaths, of which 344 (9.74%) was attributed to medical outliers. A similar percentage (10.50%) was observed for 30-day mortality. Approximately 11% of the total readmissions to the hospital, was to patients that were medical outliers at some point during their care. Univariate analysis of medical outliers on the patient outcome revealed that medical outliers are not associated with in-hospital or 30-day mortality but they do affect the readmission probabilities with statistically significant odds ratio (95% CI) of 1.193 (1.110 – 1.282).

Table 2: Patient outcomes and medical outliers

Characteristic	Non-outlying	Out-lying	Effect size (95% CI)**	P-value

**In-hospital mortality**	3,187 (90.26)	344 (9.74)	0.983 (0.877 -1.102)	0.773
**30-day mortality**	2,072 (89.50)	243 (10.50)	1.071 (0.936 – 1.227)	0.315
**Readmission**	7,592 (88.66)	971 (11.34)	1.193 (1.110 – 1.282)	<0.001
**LOS***	3(7)	7(7)	0.672 (0.624 – 0.719)	<0.001

All values are numbers (percentages) unless otherwise stated
*Median (IQR)
**Odds ratio with the exception of LOS that the negative binomial regression coefficient is given

Table 2 shows that outliers have more than double the LOS of non-medical outlier spells. As the distribution of LOS is highly positively skewed, median (IQR) is reported. LOS is further described in Figure 2 where the distribution of both spell groups is shown. It is clear that medical outliers are staying in the hospital for longer periods of time and more patient spells have recorded more than 30 days in hospital compared to non-medical outliers. Univariate analysis for LOS indicated that the log count for medical outlier patients is 0.672 more than non-medical outliers, equating to staying in hospital approximately two days longer than non-medical outliers.

<<Figure 2>>

After adjusting for variables that may affect the patient outcomes we were investigating, it was found that medical outliers are not associated with increased odds of in-hospital mortality, 30 day mortality or readmission, Table 3. However, medical outliers were found to be associated with an increasing effect on LOS. Medical outliers compared to non-medical outliers, while adjusting for all other variables and keeping them constant the model, have a log count of stay of 0.042 higher compared to non-medical outliers. The expected LOS for non-medical outliers was 3.86 (95% CI 3.22 to 4.50) while for medical outliers was 4.03 (95% CI 3.36 to 4.7). In other words, medical outliers spent 0.17 days (p-value 0.006) more in hospital compared to non-medical outliers.

Table 3: Effect sizes of medical outliers on patient outcomes adjusting for other independent variables in the multivariable models

Patient Outcome	Effect size*	Significance (0.05)	(95% CI)
**In-hospital mortality**	0.679	<0.001	0.600 - 0.769
**30-day mortality**	0.711	<0.001	0.614 - 0.822
**Readmission**	0.928	0.09	0.849 - 1.013
**LOS**	0.042	0.004	0.014 - 0.071

*Odds ratio with the exception of LOS that the zero truncated negative binomial coefficient is given

For peer review only

Discussion

Our analysis showed that approximately 10% of the patients classified as medical patients were outliers at least once during their entire hospitalisation. The results indicated that age, comorbidity, emergency admissions within the same year, primary diagnosis category and weekend admission impose significant effects on in-hospital mortality, 30-day mortality, readmissions and LOS. These factors are known to affect the patient outcomes we used in this study. Being a medical outlier does not appear to affect mortality, either in-hospital or 30-day after discharge. This was also observed after controlling for confounding variables. The preventive odds observed for mortality outcomes during the multivariable analysis can be explained by the fact that patients classified based on medical expertise as being more severely affected by their disease/condition, are kept on wards designated and specific for their condition and patients that become outliers are typically less unwell.

Being a medical outlier is associated with higher odds of being readmitted to the same hospital within 30 days but after adjusting for a number of confounding variables this association was diluted. However, we found that outlier patients have double the length of stay of non-outlying patients and multivariable analysis confirmed this increase in length of stay.

Our results are in-line with those found in the extant literature comprising a limited number of studies. Specifically, Alameda and Suarez in a 2009 study performed in Spain found that readmissions were not affected by being a medical outlier but that there was an increasing effect on LOS.[13] Longer LOS for outlying patients was also observed by Stowell et al. in a French study in 2013.[18]

The increased length of stay medical outliers have compared to non-medical outliers could be explained by the difference in primary disease diagnosis. As stated previously the primary diagnosis differed between the two groups with outliers being predominantly diagnosed with circulatory and respiratory diseases whereas non-outliers were primarily diagnosed with circulatory diseases and symptoms that could not be classified. Length of stay is an important metric of quality of healthcare provision as it is often associated with complications occurring within the hospital.[30–32] Further reasons why medical outlying patients may experience longer length of stays include the delayed medical contact between arrival and first examination in the case the patient was placed straight away on an “inappropriate” ward, insufficient contact with medical and nursing personnel during outlying period and also inability of nursing personnel to meet patient needs because of lack of specialisation on the disease/condition of the patient.

For peer review only

The field of medical outliers is not extensively researched although evidence does exist to suggest that being in the right bed at the right time is beneficial to patients as a number of studies in stroke, burns, asthma and gastrointestinal haemorrhage has shown. [33–36] However, there is not enough evidence in the literature to give a definitive answer on the effect of medical outliers on patient outcomes. This is because some studies were only looking at qualitative data to understand what causes medical outliers, or because the quantitative studies were based on small sample sizes with a lot of exclusion criteria or performed in a single hospital department or ward. Our study is the first to analyse the effect medical outliers have on patient outcomes using a large set of routinely collected data not limited to a specific clinical service, in the UK.

Limitations

Limitations are inevitable in retrospective, observational studies. Our study used routinely collected data from the hospital's patient administration system, which is known to be a limiting factor in studies. The secondary data usage is of limited use for confounding factors as we could not adjust for factors we believed would be important in the multivariable models we developed. For example we could not adjust for the severity or comorbidity of a patient although, for the latter, we used the proxies of number of procedures and diagnosis codes recorded in the patient record for that spell. A prospective study, could allow for the collection of specific clinical data such as severity of the patient and other factors associated with patient movement and outlier status such as time to examine patients on ward rounds or patient complications but this would need a lot of resources to be able to get as many data.

Out study is a single centre study which in theory minimises generalisability although the amount of routinely collected data we used somewhat mitigates this limitation. Given the lack of relevant data being recorded in national datasets such as Hospital Episode Statistics (HES) only a study which includes prospective data collection method could overcome this limitation fully.

Reasons for longer patient LOS could not be investigated as they are not typically recorded in administrative databases. We could not identify nursing and medical staff numbers per ward, experience of nurses and doctors, delay in prescription of drugs or laboratory analyses and mistakes occurring during prescription or diagnosis. These could be further investigated to elucidate what makes outlying patients more susceptible to different patient outcomes compared to non-outlying patients.

Longer length of stay in hospital makes the patients susceptible to hospital acquired infections and could be further investigated. Again, infection data are not routinely collected for every patient admission.

For peer review only

Finally, our study investigated medical outliers only and not surgical or maternity cases. A recent publication by The Royal College of Surgeons of England showed that surgical patients too experience outlying, which raises the same concerns regarding patient safety, continuity of care and overall levels of provision of high quality healthcare.[10]

Conclusions

There is plenty of anecdotal and some research evidence to suggest that, in busy acute care hospitals, the phenomenon of medical outliers is commonplace. In the district general hospital that we studied using routinely collected data over a three year period, we found that almost one in 10 medical patient spells spent at least part of their stay in a non-medical ward.

After adjusting for other factors, we found medical outliers were associated with an increased LOS. We also found that mortality or re-admissions were not worse than patients treated in appropriate specialty wards. This is in line with existing, but limited literature that such patients experience worse patient outcomes. However, unlike previous studies on this issue, our study was not limited to a single clinical service.

In light of our findings, hospitals may need to revisit their policies regarding outlying patients since there is evidence suggesting that longer length of stay is associated with an increased likelihood of harm events, worse quality of care and increased healthcare costs.

However, on the balance of evidence, we cannot advise on taking steps towards eliminating the practice entirely since that may have negative implications on bed availability, which in turn may lead to increases in cancellations of elective cases and longer emergency department waiting times for hospital admission among other negative consequences.

Rather, our findings could be put in better use by informing discussions between clinicians and managers at hospital, specialty and ward levels of the negative effects associated with medical outliers. These effects should be taken into account when devising policies and procedures (e.g., in ring-fencing pools of beds for specific types of patients), or when deciding on bed management issues on an ad-hoc basis.

As it is common with secondary data analysis studies, more research is needed to investigate the effects more thoroughly through, for example, a multi-centre study of prospectively collected data. The call for additional research is particularly relevant here as the practice of placing patients on any available hospital bed appears to be wide spread.

For peer review only

Acknowledgments/ Funding

The Researchers-in-Residence programme of research was funded by the Royal United Hospitals NHS Foundation Trust.

Conflict of interest

We would like to declare that Robin Fackrell is the Head of Medical Division at the RUH Bath. Christos Vasilakis and Neophytos Stylianou both received funding form Royal United Hospitals of Bath NHS Trust to perform this research as part of the Researchers-in-Residence programme.

Contributory statement

NS, RF and CV conceived the idea for the study. NS and CV designed the study. NS analysed all data and wrote the original manuscript with input from CV. RF facilitated the acquisition of data and provided clinical expert guidance. All authors commented on the manuscript.

Data sharing statement

Data were provided by the Business Intelligence Unit of Royal United Hospitals of Bath. As we cannot keep the data based on data sharing agreements in place, data can be requested by the provider if data sharing agreement requirements are met by recipient.

References

1 Goulding L, Adamson J, Watt I, *et al.* Lost in hospital: A qualitative interview study that explores the perceptions of NHS inpatients who spent time on clinically inappropriate hospital wards. *Heal Expect* 2015;**18**:982–94. doi:10.1111/hex.12071

2 Goulding L, Adamson J, Watt I, *et al.* Patient safety in patients who occupy beds on clinically inappropriate wards: a qualitative interview study with NHS staff. *BMJ Qual Saf* 2012;**21**:218–24. doi:10.1136/bmjqs-2011-000280

3 Audit Commission. Bed management. London: 2003.

4 Wright N, Shapiro LM, Nicholson J. Treatment of renal failure in a non-specialist unit. *Br Med J* 1980;**281**:117–8.http://www.ncbi.nlm.nih.gov/pubmed/7427208 (accessed 4 Aug2016).

5 Bulman R. Bed Management Policy. Salford: 2016.

6 Cullen S, OArk C, Jones L, *et al.* Standard Operating Procedure for Outlying Patients. Kendal: 2015.

7 Martin N. Adult Outliers Policy. Portsmouth: 2015.

8 Purse M. Adult Outliers & Buffer Capacity Policy. Portsmouth: 2009.

9 Santamaria JD, Tobin AE, Anstey MH, *et al.* Do outlier inpatients experience more emergency calls in hospital? An observational cohort study. *Med J Aust* 2014;**200**:45–8. doi:10.5694/mja12.11680

For peer review only

10 Williamson M, Ghazaly M, Bhatt N. Looking after patients, not looking for patients. *Bull R Coll Surg Engl* 2015;**97**:260–2. doi:10.1308/147363515X14272809070960

11 Cornwell J, Levenson R, Sonola L, *et al.* Continuity of care for older hospital patients A call for action. London: 2012.

12 Freeman G, Hughes J. Continuity of care and the patient experience. London: 2010.

13 Alameda C, Suarez C. Clinical outcomes in medical outliers admitted to hospital with heart failure. *Eur J Intern Med* 2009;**20**:764–7. doi:10.1016/j.ejim.2009.09.010

14 Lloyd JM, Elsayed S, Majeed A, *et al.* The practice of out-lying patients is dangerous: A multicentre comparison study of nursing care provided for trauma patients. *Injury* 2005;**36**:710–3. doi:10.1016/j.injury.2004.11.006

15 Lepage B, Robert R, Lebeau M, *et al.* Use of a risk analysis method to improve care management for outlying inpatients in a university hospital. *Qual Saf Heal Care* 2009;**18**:441–5. doi:10.1136/qshc.2007.025742

16 National Audit Office. Inpatient Admissions and Bed Management in NHS acute hospitals. London: 2000. http://www.nao.org.uk/publications/9900/inpatient_admissions_and_bed.aspx

17 Serafini F, Fantin G, Brugiolo R, *et al.* Outlier admissions of medical patients: prognostic implications of outlying patients. The experience of the Hospital of Mestre. *Ital J Med* 2015;**9**:299–302. doi:10.4081/itjm.2015.528

18 Stowell A, Claret P-G, Sebbane M, *et al.* Hospital out-lying through lack of beds and its impact on care and patient outcome. *Scand J Trauma Resusc Emerg Med* 2013;**21**:17. doi:10.1186/1757-7241-21-17

19 Creamer GL, Dahl A, Perumal D, *et al.* Anatomy of the ward round: the time spent in different activities. *ANZ J Surg* 2010;**80**:930–2. doi:10.1111/j.1445-2197.2010.05522.x

20 Perimal-Lewis L, Li JY, Hakendorf PH, *et al.* Relationship between in-hospital location and outcomes of care in patients of a large general medical service. *Intern Med J* 2013;**43**:712–6. doi:10.1111/imj.12066

21 Perimal-Lewis L, Bradley C, Hakendorf PH, *et al.* The relationship between in-hospital location and outcomes of care in patients diagnosed with dementia and/or delirium diagnoses: analysis of patient journey. *BMC Geriatr* 2016;**16**:190. doi:10.1186/s12877-016-0372-5

22 Kadum D. Right Patient, Right Place. Shrewsbury: 2013.

23 Bryant M, Daly M. Right Patient, Right care, Right Place. Exeter: 2011.

24 NRES. Defining Research: NRES guidance to help you decide if your project requires review by a Research Ethics Committee. London: 2009.

25 HSCIC. NHS DATA MODEL AND DICTIONARY. http://www.datadictionary.nhs.uk/web_site_content/navigation/main_menu.asp

26 HSCIC. PbR data user guide. Leeds: 2013.

27 English indices of deprivation 2015. 2015.https://www.gov.uk/government/statistics/english-indices-of-deprivation-2015

28 ONS. Super Output Area (SOA). 2016.http://webarchive.nationalarchives.gov.uk/20160105160709/http://www.ons.gov.uk/o

For peer review only

ns/guide-method/geography/beginner-s-guide/census/super-output-areas--soas-/index.html

29 Stata: Data Analysis and Statistical Software. http://www.stata.com/

30 McAleese P, Odling-Smee W. The effect of complications on length of stay. *Ann Surg* 1994;**220**:740–4.http://www.ncbi.nlm.nih.gov/pubmed/7986140 (accessed 14 Dec2016).

31 Hassan M, Tuckman HP, Patrick RH, *et al.* Hospital length of stay and probability of acquiring infection. *Int J Pharm Healthc Mark* 2010;**4**:324–38. doi:10.1108/17506121011095182

32 Dulworth S, Pyenson B. Healthcare-associated infections and length of hospital stay in the Medicare population. *Am J Med Qual* 2004;**19**:121–7.

33 Sanderson JD, Taylor RF, Pugh S, *et al.* Specialized gastrointestinal units for the management of upper gastrointestinal haemorrhage. *Postgrad Med J* 1990;**66**:654–6.http://www.ncbi.nlm.nih.gov/pubmed/2217034 (accessed 4 Aug2016).

34 Bucknall CE, Robertson C, Moran F, *et al.* Management of asthma in hospital: a prospective audit. *Br Med J (Clin Res Ed)* 1988;**296**:1637–9.http://www.ncbi.nlm.nih.gov/pubmed/3135048 (accessed 4 Aug2016).

35 Mayor S. Stroke patients prefer care in specialist units. *BMJ* 2005;**331**:130. doi:10.1136/bmj.331.7509.130-a

36 Dunn KW. Standards and Strategy for Burn Care. 2001. http://www.ibidb.org/downloads-mainmenu-28/cat_view/14-general-reports (accessed 14 Oct2011).

Figures

Figure 1: An illustrative example of data structure and classification scheme used to define a medical outlier (Patient Spell 2 in this example). Only medical spells were considered, which are those spells with the dominant episode being allocated to a medical specialty.

Figure 2: Distribution of LOS for medical outlier and non-medical outliers

For peer review only

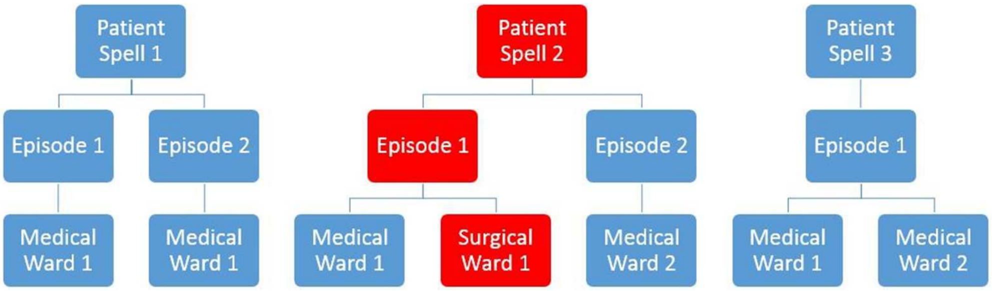

An illustrative example of data structure and classification scheme used to define a medical outlier (Patient Spell 2 in this example). Only medical spells were considered, which are those spells with the dominant episode being allocated to a medical specialty

89x25mm (300 x 300 DPI)

For peer review only

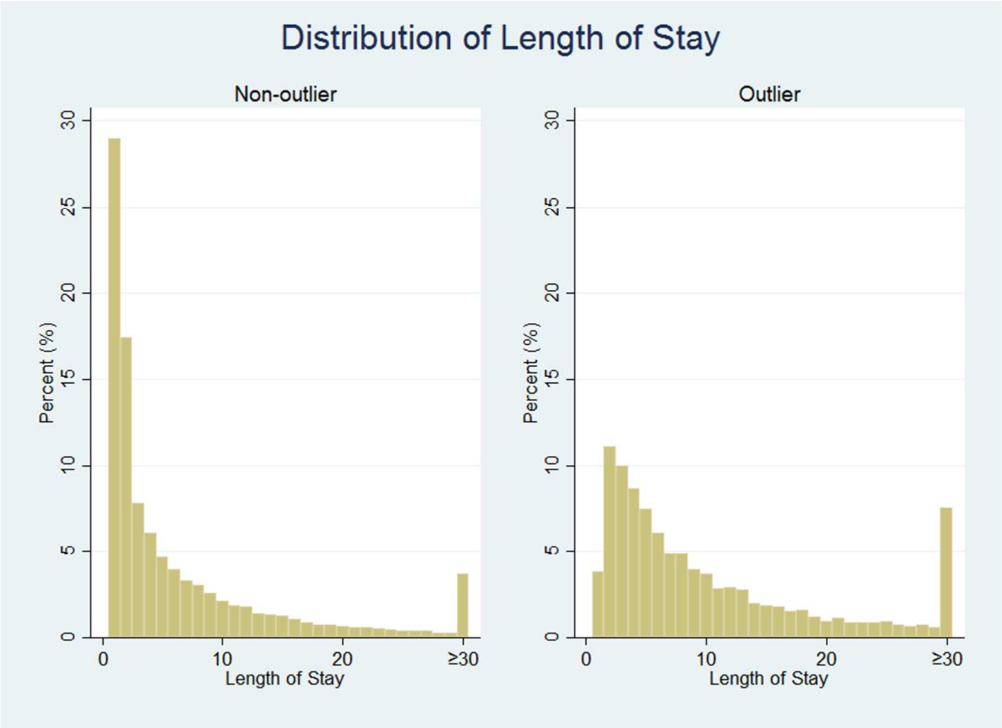

Distribution of LOS for medical outlier and non-medical outliers

75x54mm (300 x 300 DPI)

For peer review only

STROBE Statement—Checklist of items that should be included in reports of ***cross-sectional studies***

	Item No	Recommendation
**Title and abstract**	1	(*a*) Indicate the study's design with a commonly used term in the title or the abstract<br>**Within the title (page 1) and study design section of the abstract (page 2)**
		(*b*) Provide in the abstract an informative and balanced summary of what was done and what was found<br>**See principal findings and conclusion of the abstract (page 2)**
**Introduction**		
Background/rationale	2	Explain the scientific background and rationale for the investigation being reported<br>**See introduction section of paper (page 4-5)**
Objectives	3	State specific objectives, including any prespecified hypotheses<br>**See introduction section of paper (page 5)**
**Methods**		
Study design	4	Present key elements of study design early in the paper<br>**Methods section (page 5)**
Setting	5	Describe the setting, locations, and relevant dates, including periods of recruitment, exposure, follow-up, and data collection<br>**Methods section (page 5)**
Participants	6	(*a*) Give the eligibility criteria, and the sources and methods of selection of participants
Variables	7	Clearly define all outcomes, exposures, predictors, potential confounders, and effect modifiers. Give diagnostic criteria, if applicable<br>**Methods section (page 6)**
Data sources/ measurement	8*	For each variable of interest, give sources of data and details of methods of assessment (measurement). Describe comparability of assessment methods if there is more than one group
Bias	9	Describe any efforts to address potential sources of bias<br>**Methods section (page 6)**
Study size	10	Explain how the study size was arrived at<br>**(N/A)**
Quantitative variables	11	Explain how quantitative variables were handled in the analyses. If applicable, describe which groupings were chosen and why<br>**Methods section (pages 5-6)**
Statistical methods	12	(*a*) Describe all statistical methods, including those used to control for confounding<br>**Methods section (page 6)**
		(*b*) Describe any methods used to examine subgroups and interactions
		(*c*) Explain how missing data were addressed<br>**(N/A)**
		(*d*) If applicable, describe analytical methods taking account of sampling strategy<br>**(N/A)**
		(*e*) Describe any sensitivity analyses<br>**(N/A)**
**Results**		
Participants	13*	(a) Report numbers of individuals at each stage of study—eg numbers potentially eligible, examined for eligibility, confirmed eligible, included in the study, completing follow-up, and analysed

For peer review only

		(b) Give reasons for non-participation at each stage
		(c) Consider use of a flow diagram **Results section table 1 (page 7)**
Descriptive data	14*	(a) Give characteristics of study participants (eg demographic, clinical, social) and information on exposures and potential confounders **Results section table 1 (page 7)**
		(b) Indicate number of participants with missing data for each variable of interest **(N/A)**
Outcome data	**15***	**Report numbers of outcome events or summary measures** **Results section Table 2 (page 8)**
Main results	16	(*a*) Give unadjusted estimates and, if applicable, confounder-adjusted estimates and their precision (eg, 95% confidence interval). Make clear which confounders were adjusted for and why they were included **Results section/ tables 2 & 3**
		(*b*) Report category boundaries when continuous variables were categorized **(N/A)**
		(*c*) If relevant, consider translating estimates of relative risk into absolute risk for a meaningful time period **(N/A)**
Other analyses	17	Report other analyses done—eg analyses of subgroups and interactions, and sensitivity analyses **(N/A)**
**Discussion**		
Key results	18	Summarise key results with reference to study objectives **Discussion section first paragraph (page)**
Limitations	19	Discuss limitations of the study, taking into account sources of potential bias or imprecision. Discuss both direction and magnitude of any potential bias **Discussion section of the paper/Limitation sub-section (page 10)**
Interpretation	20	Give a cautious overall interpretation of results considering objectives, limitations, multiplicity of analyses, results from similar studies, and other relevant evidence **Discussion and conclusion section of paper (pages 9-11)**
Generalisability	21	Discuss the generalisability (external validity) of the study results **Discussion section of the paper (page 11)**
**Other information**		
Funding	22	Give the source of funding and the role of the funders for the present study and, if applicable, for the original study on which the present article is based **Within acknowledgements (page 12)**

*Give information separately for exposed and unexposed groups.

Note: An Explanation and Elaboration article discusses each checklist item and gives methodological background and published examples of transparent reporting. The STROBE checklist is best used in conjunction with this article (freely available on the Web sites of PLoS Medicine at http://www.plosmedicine.org/, Annals of Internal Medicine at http://www.annals.org/, and Epidemiology at http://www.epidem.com/). Information on the STROBE Initiative is available at www.strobe-statement.org.

For peer review only

For peer review only - http://bmjopen.bmj.com/site/about/guidelines.xhtml
